# Risk Factors for Postoperative Acute Kidney Injury Requiring Renal Replacement Therapy in Patients Undergoing Heart Valve Surgery

**DOI:** 10.3390/jcm13247811

**Published:** 2024-12-20

**Authors:** Piotr Duchnowski, Witold Śmigielski

**Affiliations:** Cardinal Wyszynski National Institute of Cardiology, Alpejska 42, 04-628 Warsaw, Poland; wsmigielski@ikard.pl

**Keywords:** acute kidney injury (AKI), renal replacement therapy (RRT), heart valve surgery, postoperative complications, C-reactive protein (CRP), Troponin T, markers of myocardial damage

## Abstract

**Background:** Postoperative acute kidney injury (AKI) in patients undergoing heart valve surgery is a common complication requiring special treatment, including renal replacement therapy (RRT). Effective prevention remains the most effective tool to reduce this important clinical problem. The aim of the study was to evaluate the predictive abilities of selected perioperative parameters in predicting AKI requiring RRT in the early postoperative period in patients undergoing cardiac valve surgery. **Methods:** Prospective study on a group of patients undergoing cardiac valve surgery. The primary endpoint was postoperative AKI requiring RRT. The secondary endpoint was death in the RRT group. Logistic regression analysis was used to assess which variables predicted the primary and secondary endpoints. **Results:** 603 patients were included in the study. The primary endpoint occurred in 43 patients. At multivariable analysis, age (*p* < 0.001), preoperative CRP level (*p* = 0.007), troponin T measured one day after surgery (TnT II) (*p* < 0.001) and prolonged postoperative use of catecholamines (*p* = 0.001) were independent predictors of the primary endpoint. In turn, death in the group of patients requiring RRT occurred in 32 patients. Age (*p* < 0.001), preoperative CRP level (*p* = 0.002), TnT II (*p* = 0.009), and prolonged postoperative use of catecholamines (*p* = 0.001) remained independent predictors of the secondary endpoint. **Conclusions:** The results of this study indicate that older age, elevated values of preoperative levels of CRP, as well as increasing levels of postoperative troponin T and the need for a prolonged supply of catecholamines, are independent predictors of postoperative AKI requiring RRT as well as death. Accurate identification of patients at increased postoperative risk of AKI could facilitate preoperative patient informed consent and optimize the process of qualification and cardiac surgical treatment.

## 1. Introduction

Acute kidney injury (AKI) occurring in the early period after open heart surgery is a complication that extends the hospitalization period, increases the use of hospital resources, and significantly affects short- and long-term mortality. The incidence of AKI in the postoperative period is high and ranges from 20% to 70% of cases. Severe forms of AKI requiring renal replacement therapy (RRT) are less common but are characterized by a high mortality rate, ranging from 40% to 70% [1,2,3,4,5,6,7,8]. An acute kidney injury (AKI) is a clinical syndrome diagnosed when the serum creatinine concentration increases above 0.3 mg/dL in 48 h or more than 1.5 times in the last seven days or when urine output < 0.5 mL/kg/h for the next six hours [9,10,11]. Traditionally, three types of acute kidney injury are distinguished: prerenal, parenchymal and postrenal. Prerenal acute kidney injury is mainly a consequence of impaired renal perfusion resulting from a reduction in effective circulating blood volume (hypovolemia) as well as low cardiac output. The available literature describes several mechanisms responsible for the development of AKI in patients undergoing cardiac surgery, including: renal hypoperfusion (heart failure, use of extracorporeal circulation, hemorrhages during surgery), inflammation and oxidative stress (tissue damage during surgery, blood flow through the cardiopulmonary bypass circuit), the use of drugs and nephrotoxic agents such as angiotensin-converting enzyme inhibitors (ACEI), angiotensin receptor blockers (ARB), non-steroidal anti-inflammatory drugs (NSAIDs) or exposure to contrast agents, as well as embolic factors and genetic predisposition [1,12,13,14,15,16,17,18,19]. In recent years, in various areas of cardiology, especially in diagnostics and prognosis, two important markers of myocardial damage have been widely used, i.e., troponin T (TnT) determined by high-sensitivity tests and N-terminal natriuretic peptide (NT-proBNP). The use of the above parameters is now the daily practice of every cardiologist [20,21]. Due to the high risk of death and limited further therapeutic options in patients with RRT, the main aim of the present study was to evaluate selected perioperative parameters, including markers of myocardial damage, in predicting postoperative RRT in the early postoperative period in patients undergoing heart valve surgery.

## 2. Methods

A prospective study conducted at the Cardinal Wyszyński National Institute of Cardiology in Warsaw on a group of patients undergoing heart valve surgery with or without concomitant coronary artery disease. The exclusion criteria for participation in the study were: lack of consent to participate in the study, patient’s age below 18 years, presence of active cancer, preoperative end-stage renal failure requiring renal replacement therapy, presence of significant stenosis in the carotid artery and presence of a porcelain aorta. On the day before cardiac surgery, on postoperative day zero and on the first day after surgery, blood was collected from each patient to determine the level of biomarkers. The Sysmex K-4500 automatic analyzer (Sysmex, Kobe, Japan) was used to determine individual blood morphological parameters. NT-proBNP concentration was determined using Elecsys 2010 electrochemiluminescent immunoassays (Roche, Munich, Germany). The level of high sensitivity troponin T in plasma was determined using Troponin hs-STAT (Roche). The cardiac surgery was performed under general anesthesia using cardiopulmonary bypass and aortic cross-clamping. The primary endpoint was acute kidney injury (AKI), which was diagnosed in the early perioperative period and required renal replacement therapy in the form of continuous venovenous hemodiafiltration. The diagnosis of acute kidney injury was made when the serum creatinine concentration increased >0.3 mg/dl in 48 h or >1.5-fold in the last 7 days and/or a decrease in diuresis < 0.5 mL/kg/h for 6 h. In each patient, in the first days after cardiac surgery, creatinine concentration and daily urine collection were assessed daily. The decision to use renal replacement therapy was made by the teams of anesthesiologists caring for patients with AKI based on changes in laboratory test results and the patient’s clinical condition. The secondary endpoint was death from any cause in the group of patients with postoperative AKI requiring RRT. In each case, early postoperative observation of patients was carried out in the intensive care unit, and then, in most cases, after stabilization of the patient’s clinical condition, in the cardiac surgery department, until discharge from the hospital or until death occurred. The protocol of the presented study was approved by the Bioethics Committee of the National Institute of Cardiology in Warsaw—Study No. 2.32/VI/18; approval date 14 May 2018). Each patient included in the study provided written consent before enrollment in the study.

### Statistical Analysis

STATISTICA 12 software (StatSoft Polska Sp. z o. o.; Kraków, Poland) was used to perform statistical analyses. Data were presented using median (Q1–Q3) and frequency (%). Intergroup cooperation was performed using the Mann–Whitney U test for quantitative variables and the chi-square test for qualitative variables. Logistic regression analysis was used to determine the predictors of the primary and secondary endpoints. Statistically significant variables obtained in univariate analysis were used to conduct multivariate logistic regression analysis. The significance level was set as *p* < 0.05. To assess the predictive ability of the age, CRP, and troponin t measured one day after surgery (TnT II), receiver operating characteristic (ROC) curves analysis was used.

## 3. Results

**Figure 1:**
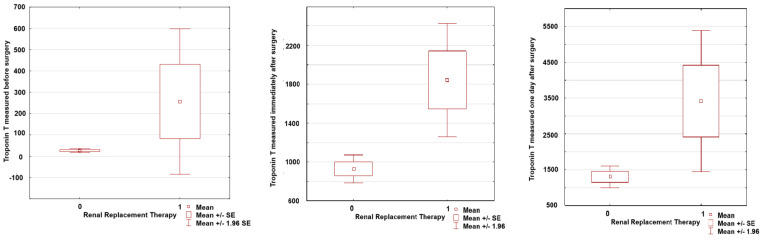
Troponin T values determined at successive time points stratified by primary endpoint.

The study included 603 patients who underwent cardiac surgery for valve repair/replacement at Cardinal Wyszynski National Institute of Cardiology, Warsaw, Poland. The characteristics of the patients divided into patients with and without the primary endpoint are presented in Table 1. The average age was 65 (57–71) years. A total of 58% of patients included in the study were men. The primary endpoint occurred in 43 patients. Among the patients requiring RRT, 39 patients (90% of patients with RRT) required prolonged supply of catecholamines due to postoperative hemodynamic instability, including 11 patients who required the use of mechanical circulatory support (MCS) due to the development of full-blown cardiogenic shock, and 34 patients developed multiple organ dysfunction syndrome (MODS). Factors predicting the occurrence of the primary endpoint are presented in Table 2. At multivariable analysis age (OR 1.084; 95% CI 1.031–1.114; *p* < 0.01), preoperative CRP level (OR 1.727; 95% CI 1.157–2.576; *p* = 0.007), troponin T measured one day after surgery (TnT II) (OR 2.058; 95% CI 1.361–3.113; *p* < 0.001) and prolonged postoperative use of catecholamines (OR 5.399; 95% CI 1.973–14.770; *p* = 0.001) were independent predictors of the primary endpoint. Receiver operating characteristic analysis established an age cutoff of 73 years, preoperative CRP of 0.18 mg/dL, and TnT II of 596 ng/L to predict the primary endpoint. Figure 1 shows troponin T values determined at successive time points, stratified by primary endpoint. Patients in the RRT group more often required prolonged supply of catecholamines due to the development of postoperative hemodynamic instability (MCS), rethoracotomy, among others, due to postoperative bleeding; multi-organ failure syndrome was more common, they stayed longer in the intensive care unit, and they had a significantly higher 30-day mortality rate.

The secondary endpoint occurred in 32 patients. Factors predicting death in the RRT group are presented in Table 3. At multivariable analysis age (OR 1.118; 95% CI 1.049–1.328; *p* < 0.01), preoperative CRP level (OR 2.017; 95% CI 1.278–3.181; *p* = 0.002), TnT II (OR 1.830; 95% CI 1.161–2.883; *p* = 0.009) and prolonged postoperative use of catecholamines (OR 7.618; 95% CI 2.181–26.610; *p* = 0.001) remained independent predictors of the secondary endpoint. Receiver operating characteristic analysis established an age cutoff of 71 years, preoperative CRP of 0.46 mg/dL, and TnT II of 1367 ng/L to predict the secondary endpoint. The main cause of death among RRT patients was multi-organ failure syndrome.

## 4. Discussion

Postoperative AKI in the group of patients undergoing cardiac surgery due to valvular heart disease is a common complication requiring special treatment, including the use of RRT. Effective prevention therefore remains the most effective tool to reduce this important clinical problem [22,23,24]. Optimization of the preventive strategy is possible by determining appropriate preoperative and perioperative predictive factors. In our study, we showed that AKI requiring renal replacement therapy occurred in 43 patients out of 603 included in the study, which constituted approximately 7 percent of patients. Predictors of postoperative RRT included age, preoperative CRP level, troponin T level measured on the first postoperative day, and the need for prolonged supply (over 48 h) of catecholamines from the end of surgery. It is worth noting that in the univariate analysis, markers of myocardial damage such as preoperative levels of troponin T and NT-proNBP, as well as the troponin T level measured immediately after surgery, were also predictive factors for the occurrence of the primary endpoint. The authors also showed that the primary endpoint was significantly more common in patients with a higher EuroSCORE II calculation score as well as lower preoperative hemoglobin values.

One of the main determinants of the body’s ability to deliver oxygen to body cells, apart from blood morphotic parameters, is cardiac output (CO), defined as the amount of blood pumped by the left ventricle in one minute [25]. Heart failure, characterized by, among other things, low cardiac output, is a common phenomenon among patients admitted to the cardiac intensive care unit after heart valve surgery. The available literature shows that as many as over 50% of patients undergoing cardiac surgery receive catecholamines in the early postoperative period, mainly due to postoperative low cardiac output syndrome (LCOS) [26,27]. In the present study, 39 out of 43 patients with RRT required prolonged administration of catecholamines for more than 48 h due to persistent postoperative hemodynamic instability. NT-proBNP and TnT are important markers of myocardial damage, the importance of which has been unquestionable in recent decades in the diagnosis and prognosis of many cardiological entities, including heart failure [21]. The left ventricular muscle of a patient with severe symptomatic valvular disease is overloaded by increased blood pressure and/or increased blood volume, which forces increased secretion of NT-proBNP and TnT by cardiomyocytes. Long-term overload of the left ventricular muscle promotes degenerative processes in the left ventricular muscle, including gradual necrosis and fibrosis of cardiomyocytes [28,29]. Therefore, elevated preoperative NT-proBNP and TnT values in the blood of patients with severe valvular heart disease may indicate significant overload or even decompensation of the left ventricular muscle [30]. The above may therefore suggest that the overloaded heart muscle is particularly sensitive to non-physiological conditions prevailing in the perioperative period, related to, among others, the use of cardioplegia, extracorporeal circulation, or blood loss, which may result in further damage to the heart muscle (which may be indicated by increasing levels of troponin T in the postoperative period) manifesting itself, among others, in postoperative hemodynamic instability and the need for a prolonged supply of catecholamines and, at the same time, lead to impaired renal blood supply and thus the development of AKI.

The study showed that out of 43 patients with postoperative AKI requiring renal therapy, death occurred in as many as 32 patients. Importantly, multivariate analysis showed that the factors predicting death in patients with RRT were age, preoperative CRP, troponin T measured on day 1 after surgery, and the need for prolonged supply of catecholamines. The study results indicate that, in addition to parameters indicating myocardial damage, CRP and age are important for the occurrence of AKI requiring RRT and postoperative death. With age, the human body’s susceptibility to the destructive effects of intra- and extra-environmental factors increases, and its adaptive abilities decrease, which is a consequence of reduced physiological reserves of many organs [31]. One of the factors responsible for this process may be chronic inflammation. CRP is a cyclic pentamer belonging to the pentaxin family-proteins that bind ligands in calcium-dependent reactions. CRP synthesis takes place primarily in the liver in response to pro-inflammatory factors: Interleukin-1 (IL-1), Interleukin-6 (IL-6). CRP is one of the most important acute phase proteins, whose role is to participate in the body’s immune response by facilitating complement fixation. The available literature shows that increased CRP and IL-6 values are predictors of worse physical fitness and cognitive functions. So far, it has been described that elevated CRP values are influenced by factors such as diabetes, a sedentary lifestyle, smoking, and obesity. Numerous studies have also documented that chronic inflammation is associated with increased morbidity and mortality [32,33].

Therefore, to sum up, taking into account the fact that AKI requiring RRT is a dynamic complication, most often developing in the first postoperative days, the results of our study indicate the need for close monitoring of patients from the moment of completion of cardiac surgery. The group particularly exposed to the development of AKI requiring the use of RRT and death are primarily hemodynamically unstable patients requiring the supply of catecholamines, with elevated preoperative and postoperative values of markers of cardiac damage, such as troponin T and NT-proBNP, as well as with elevated values of preoperative CRP levels and in old age.

### Limitations of the Study

This was a single-center study with a limited number of patients and a limited number of endpoints. There is therefore a risk of overfitting the statistical model. Enlarging the group and increasing the number of centers in the study may confirm the obtained results.

## 5. Conclusions

The results of this study indicate that age, elevated values of preoperative levels of CRP, as well as increasing levels of postoperative troponin T and the need for a prolonged supply of catecholamines, are independent predictors of postoperative AKI requiring the implementation of renal replacement therapy as well as death in the early postoperative period in patients undergoing heart valve surgery. Accurate identification of patients at increased postoperative risk of AKI could facilitate preoperative patient informed consent and optimize the process of qualification and cardiac surgical treatment.

## Figures and Tables

**Table 1:** Baseline characteristics of the study population (*n* = 603).

Preoperative Characteristics of Patients	Values All Patients (*n* = 603)	Values Patients with RRT (*n* = 43)	Values Patients Without RRT (*n* = 560)	*p*-Value
Age, years	65 (57–71)	70 (59–76)	65 (56–70)	<0.001
Male: men, *n* (%)	353 (58)	23 (53)	330 (58)	0.59
LV ejection fraction, (%)	60 (50–65)	58 (45–65)	60 (50–65)	0.38
EuroSCORE II, %	2.4 (1.4–3.9)	3.5 (2.4–5.5)	2.35 (1.35–3.8)	<0.001
Atrial fibrillation, *n* (%)	235 (38)	27 (63)	208 (37)	<0.001
Previous myocardial infarction, *n* (%)	44 (7)	4 (9)	40 (7)	0.59
Diabetes mellitus, *n* (%)	93 (15)	9 (21)	84 (15)	0.29
Hemoglobin, g/dL	13.7 (12.7–14.7)	12.8 (10.8–13.2)	13.8 (12.8–14.7)	<0.001
GFR, mL/min/1.73 m^2^, *n* (%)	67 (55–81)	54 (36–64)	68 (57–84)	<0.001
Creatinine, [mmol/L]	68 (46–93)	118 (90–142)	65 (45–92)	<0.001
TnT, ng/L	14.4 (8–33)	24 (11.7–36)	12(7–21)	<0.001
Nt-proBNP, pg/mL	924 (321–2239)	2951 (934–4480)	855 (294–1939)	<0.001
CRP, mg/dL	0.3 (0.1–0.5)	0.4 (0.2–0.6)	0.2 (0.1–0.4)	<0.001
Aortic cross-clamp time, min	87 (60–131)	140 (78–155)	87 (60–129)	ns
Cardiopulmonary bypass time, min	114 (83–169)	190 (115–215)	113 (79–160)	0.055
Postoperative characteristics of patients				
Prolonged Postoperative Use of Catecholamines (more than 48 h), *n* (%)	202 (33)	39 (90)	163 (29)	<0.001
Mechanical circulatory support, *n* (%)	22 (4)	11 (25)	11 (2)	<0.001
Multiple organ dysfunction syndrome, *n* (%)	40 (6)	34 (79)	6 (1)	<0.001
Postoperative stroke, *n* (%)	19 (3)	11 (25)	8 (1)	<0.001
Hospital stay after surgery, day	11 (8–17)	28 (12–36)	10 (8–16)	<0.001
ICU, day	9 (8–14)	17 (6–30)	3 (2–5)	<0.001
Re-sternotomy, *n* (%)	80 (13)	9 (21)	71 (12)	0.12
30-day mortality, *n* (%)	29 (4.8)	18 (41)	11 (1,9)	<0.001
TnT II, ng/L	732 (387–1501)	2011 (952–2670)	648 (366–1261)	<0.001
TnT I, ng/L	645 (361–1121)	1262 (712–2223)	599 (345–976)	<0.001
Main procedures				
AVR, *n* (%)	321 (53)	15 (34)	306 (54)	0.01
AVP, *n* (%)	10 (2)	3 (2)	9 (2)	0.72
AVR + MVR, *n* (%)	55 (9)	12 (28)	43 (8)	<0.001
AVR + MVP, *n* (%)	11 (2)	0 (0)	11 (2)	0.35
AVP + MVP, *n* (%)	3 (0.5)	0 (0)	3 (0.5)	0.5
MVP, *n* (%)	97 (16)	11 (25)	88 (15)	0.36
MVR, *n* (%)	107 (17)	6 (14)	101 (18)	0.49
Concomitant procedure				
CABG, *n* (%)	88 (15)	11 (25)	74 (13)	0.02
TVR, *n* (%)	3 (0,5)	0 (0)	3 (0.5)	0.63
TVP, *n* (%)	159 (26)	17 (39)	142 (25)	0.04

The values are represented by median (Q1–Q3) and frequency (%). Abbreviations: AVP = aortic valve plasty, AVR = aortic valve replacement, CABG = coronary artery bypass graft, CRP = C-reactive protein, GFR = glomerular filtration rate, ICU-intensive care unit, LV = left ventricle, MVP = mitral valve plasty, MVR = mitral valve replacement, Nt-proBNP = *n*-terminal of the prohormone brain natriuretic peptide, RRT = renal replacement therapy, TnT = high sensitivity troponin t measured before surgery, TnT I = high-sensitivity troponin t measured immediately after surgery, TnT II = high-sensitivity troponin t measured one day after surgery, TVR = tricuspid valve replacement, TVP = tricuspid valve plasty.

**Table 2:** Analysis of predictive factors for the occurrence of primary endpoint.

	Univariate Analysis			Multivariate Analysis		
Variable	Odds Ratio	95% Cl	*p*-Value	Odds Ratio	95% Cl	*p*-Value
Age, years	1.073	1.035–1.112	<0.001	1.084	1.031–1.1140	<0.001
GFR < 60 mL/min/1.73 m^2^, *n* (%)	0.955	0.937–0.973	<0.001			
Hemoglobin, g/dL	0.642	0.532–0.776	<0.001			
NT-proBNP, pg/mL	2.241	1.695–2.961	<0.001			
CRP, mg/dL	2.069	1.517–2.822	<0.001	1.727	1.157–2.576	0.007
EuroSCORE II, %	1.168	1.093–1.249	<0.001			
Atrial fibrillation, *n* (%)	2.855	1.501–5.431	0.001			
Prolonged Postoperative Use of Catecholamines (more than 48 h), *n* (%)	14.378	5.972–34-612	<0.001	5.399	1.973–14.770	0.001
TnT, ng/L	2.063	1.541–2.761	<0.001			
TnT I, ng/L	2.500	1.745–3.583	<0.001			
TnT II, ng/L	2.401	1.768–3.261	<0.001	2.058	1.361–3.113	<0.001

Abbreviations: CRP = C-reactive protein, GFR = glomerular filtration rate, NT-proBNP = *n*-terminal of the prohormone brain natriuretic peptide, TnT = high sensitivity troponin t measured before surgery, TnT I = high-sensitivity troponin t measured immediately after surgery, TnT II = high-sensitivity troponin t measured one day after surgery.

**Table 3:** Analysis of predictive factors for the occurrence of secondary endpoint.

	Univariate Analysis			Multivariate Analysis		
Variable	Odds Ratio	95% Cl	*p*-Value	Odds Ratio	95% Cl	*p*-Value
Age, years	1.097	1.050–1.147	<0.001	1.118	1.049–1.328	<0.001
GFR < 60 mL/min/1.73 m^2^, *n* (%)	0.954	0.934–0.975	<0.001			
Hemoglobin, g/dL	0.625	0.505–0.774	<0.001			
NT-proBNP, pg/mL	2.271	1.655–3.118	<0.001			
CRP, mg/dL	2.236	1.585–3.156	<0.001	2.017	1.278–3.181	0.002
EuroSCORE II, %	1.153	1.079–1.232	<0.001			
Atrial fibrillation, *n* (%)	3.715	1.723–8.008	<0.001			
Prolonged Postoperative Use of Catecholamines (more than 48 h), *n* (%)	21.468	6.480–71.121	<0.001	7.618	2.181–26.610	0.001
TnT, ng/L	2.193	1.602–3.001	<0.001			
TnT I, ng/L	2.609	1.754–3.881	<0.001			
TnT II, ng/L	2.390	1.721–3.331	<0.001	1.830	1.161–2.883	0.009

Abbreviations: CRP = C-reactive protein, GFR = glomerular filtration rate, NT-proBNP = *n*-terminal of the prohormone brain natriuretic peptide, TnT = high sensitivity troponin t measured before surgery, TnT I = high-sensitivity troponin t measured immediately after surgery, TnT II = high-sensitivity troponin t measured one day after surgery.

## Data Availability

The original contributions presented in this study are included in the article. Further inquiries can be directed to the corresponding author.

## References

[1] Cheruku S.R., Raphael J., Neyra J.A., Fox A.A. (2023). Acute Kidney Injury after Cardiac Surgery: Prediction, Prevention, and Management. Anesthesiology.

[2] Tinica G., Brinza C., Covic A., Popa I.V., Tarus A., Bacusca A.E., Burlacu A. (2020). Determinants of acute kidney injury after cardiac surgery: A systematic review. Rev. Cardiovasc. Med..

[3] Hu J., Chen R., Liu S., Yu X., Zou J., Ding X. (2016). Global incidence and outcomes of adult patients with acute kidney injury after cardiac surgery: A systematic review and meta-analysis. J. Cardiothorac. Vasc. Anesth..

[4] Vives M., Hernandez A., Parramon F., Estanyol N., Pardina B., Muñoz A., Alvarez P., Hernandez C. (2020). Acute kidney injury after cardiac surgery: Prevalence, impact and management challenges. Int. J. Nephrol. Renov. Dis..

[5] Nadim M.K., Forni L.G., Bihorac A., Hobson C., Koyner J.L., Shaw A., Arnaoutakis G.J., Ding X., Engelman D.T., Gasparovic H. (2018). Cardiac and Vascular Surgery-Associated Acute Kidney Injury: The 20th International Consensus Conference of the ADQI (Acute Disease Quality Initiative) Group. J. Am. Heart Assoc..

[6] Mariscalco G., Lorusso R., Dominici C., Renzulli A., Sala A. (2011). Acute kidney injury: A relevant complication after cardiac surgery. Ann. Thorac. Surg..

[7] Wang Y., Bellomo R. (2017). Cardiac surgery-associated acute kidney injury: Risk factors, pathophysiology and treatment. Nat. Rev. Nephrol..

[8] Tecson K.M., Brown D., Choi J.W., Feghali G., Gonzalez-Stawinski G.V., Hamman B.L., Hebeler R., Lander S.R., Lima B., Potluri S. (2018). Major adverse renal and cardiac events after coronary angiography and cardiac surgery. Ann. Thorac. Surg..

[9] Khwaja A. (2012). KDIGO clinical practice guidelines for acute kidney injury. Nephron Clin. Pract..

[10] McIlroy D., Bellomo R., Billings F., Karkouti K., Prowle J., Shaw A., Myles P. (2018). Systematic review and consensus definitions for the standardised endpoints in perioperative medicine (StEP) initiative: Renal endpoints. Br. J. Anaesth..

[11] Peng K., McIlroy D.R., Bollen B.A., Billings F.T., Zarbock A., Popescu W.M., Fox A.A., Shore-Lesserson L., Zhou S., Geube M.A. (2022). Society of Cardiovascular Anesthesiologists clinical practice update for management of acute kidney injury associated with cardiac surgery. Anesth. Analg..

[12] Ronco C., Bellomo R., Kellum J.A. (2019). Acute kidney injury. Lancet.

[13] Massoth C., Zarbock A., Meersch M. (2021). Acute kidney injury in cardiac surgery. Crit. Care Clin..

[14] Andersson L., Bratteby L., Ekroth R., Hallhagen S., Joachimsson P., Van der Linden J., Wesslén O. (1994). Renal function during cardiopulmonary bypass: Influence of pump flow and systemic blood pressure. Eur. J. Cardiothorac. Surg..

[15] Lannemyr L., Bragadottir G., Krumbholz V., Redfors B., Sellgren J., Ricksten S.-E. (2017). Effects of cardiopulmonary bypass on renal perfusion, filtration, and oxygenation in patients undergoing cardiac surgery. Anesthesiology.

[16] Karim H.M.R., Yunus M., Saikia M.K., Kalita J.P., Mandal M. (2017). Incidence and progression of cardiac surgery-associated acute kidney injury and its relationship with bypass and cross clamp time. Ann. Card. Anaesth..

[17] Ranucci M., Romitti F., Isgrò G., Cotza M., Brozzi S., Boncilli A., Ditta A. (2005). Oxygen delivery during cardiopulmonary bypass and acute renal failure after coronary operations. Ann. Thorac. Surg..

[18] Sgouralis I., Evans R.G., Gardiner B.S., Smith J.A., Fry B.C., Layton A.T. (2015). Renal hemodynamics, function, and oxygenation during cardiac surgery performed on cardiopulmonary bypass: A modeling study. Physiol. Rep..

[19] Clifford K.M., Selby A.R., Reveles K.R., Teng C., Hall R.G., McCarrell J., Alvarez C.A. (2022). The risk and clinical implications of antibiotic-associated acute kidney injury: A review of the clinical data for agents with signals from the Food and Drug Administration’s Adverse Event Reporting System (FAERS) Database. Antibiotics.

[20] Duchnowski P., Śmigielski W. (2024). Usefulness of myocardial damage biomarkers in predicting cardiogenic shock in patients undergoing heart valve surgery. Kardiol. Pol..

[21] McDonagh T.A., Metra M., Adamo M. (2021). 2021 ESC Guidelines for the diagnosis and treatment of acute and chronic heart failure: Developed by the Task Force for the diagnosis and treatment of acute and chronic heart failure of the European Society of Cardiology (ESC) with the special contribution of the Heart Failure Association (HFA) of the ESC. Eur. J. Heart Fail..

[22] Silverton N.A., Lofgren L.R., Hall I.E., Stoddard G.J., Melendez N.P., Van Tienderen M., Shumway S., Stringer B.J., Kang W.-S., Lybbert C. (2021). Noninvasive urine oxygen monitoring and the risk of acute kidney injury in cardiac surgery. Anesthesiology.

[23] A Peng X., Zhu T., Chen Q., Zhang Y., Zhou R., Li K., Hao X. (2024). Simple machine learning model for the prediction of acute kidney injury following noncardiac surgery in geriatric patients: A prospective cohort study. BMC Geriatr..

[24] Wald R., Beaubien-Souligny W., Chanchlani R., Clark E.G., Neyra J.A., Ostermann M., Silver S.A., Vaara S., Zarbock A., Bagshaw S.M. (2022). Delivering optimal renal replacement therapy to critically ill patients with acute kidney injury. Intensive Care Med..

[25] Desai A.S., Jarcho A. (2017). Levosimendan for the Low Cardiac Output Syndrome after Cardiac Surgery. N. Engl. J. Med..

[26] Denault A.Y., Deschamps A., Couture P. (2010). Intraoperative hemodynamic instability during and after separation from cardiopulmonary bypass. Semin. Cardiothorac. Vasc. Anesth..

[27] Carrara A., Peluso L., Baccanelli F., Parrinello M., Santarpino G., Giroletti L., Graniero A., Agnino A., Albano G. (2024). Relationship between Preoperative Red Cell Distribution Width and Prolonged Postoperative Use of Catecholamines in Minimally Invasive Mitral Valve Surgery Patients: A Retrospective Cohort Study. J. Clin. Med..

[28] Weidemann F., Herrmann S., Störk S., Niemann M., Frantz S., Lange V., Beer M., Gattenlohner S., Voelker W., Ertl G. (2009). Impact of myocardial fibrosis in patients with symptomatic severe aortic stenosis. Circulation.

[29] Chin C.W., Shah A.S., McAllister D.A., Joanna Cowell S., Alam S., Langrish J.P., Strachan F.E., Hunter A.L., Choy A.M., Lang C.C. (2014). High-sensitivity troponin I concentrations are a marker of an advanced hypertrophic response and adverse outcomes in patients with aortic stenosis. Eur. Heart J..

[30] Kočková R., Línková H., Hlubocká Z., Mědílek K., Tuna M., Vojáček J., Skalský I., Černý Š., Malý J., Hlubocký J. (2022). Multiparametric Strategy to Predict Early Disease Decompensation in Asymptomatic Severe Aortic Regurgitation. Circ. Cardiovasc. Imaging.

[31] Ferruci L., Guralnik J.M., Studenski S., Fried L.P., Cutler G.B., Walston J.D., The Interventions on Frailty Working Group (2004). Designing randomized aimed at preventing or delaying functional decline and disability in frail, older persons: A consensus report. J. Am. Geriatr. Soc..

[32] Velissaris D., Pantzaris N., Koniari I., Koutsogiannis N., Karamouzos V., Kotroni I., Skroumpelou A., Ellul J. (2017). C-Reactive protein and frailty in the elderly: A literature review. J. Clin. Med. Res..

[33] Bruunsgaard H. (2006). The clinical impact of systemic low-level inflammation in elderly populations. With special reference to cardiovascular disease, dementia and mortality. Dan. Med. Bull..

